# Nose-to-Brain Therapeutics in Parkinson’s Disease: Clinical Trials, Mechanisms, and Therapeutic Potential

**DOI:** 10.3390/pharmaceutics18091099

**Published:** 2026-09-01

**Authors:** Emily Cherny, Tamara Minko

**Affiliations:** Ernest Mario School of Pharmacy, Rutgers, The State University of New Jersey, Piscataway, NJ 08854, USA

**Keywords:** nose-to-brain delivery, intranasal therapy, Parkinson’s disease, apomorphine, clinical trials, neurotrophic factors, nanocarriers, pharmacokinetics, translational research, rescue therapy

## Abstract

**Background/Objectives:** Intranasal delivery is a promising noninvasive approach to enhance central nervous system drug delivery in Parkinson’s disease (PD), potentially bypassing the blood–brain barrier and minimizing gastrointestinal side effects. This review summarizes clinical trials and translational evidence for intranasal PD therapies by treatment options and intent, highlighting key challenges in efficacy, pharmacokinetics, and safety. **Methods:** We reviewed human clinical trials, preclinical and pilot studies, and pharmacokinetic investigations of intranasal therapies for PD. Comparisons to established treatments were included in context. Only PD-specific clinical studies were analyzed. Therapies were grouped by rescue, antioxidant/metabolic/hormonal, and biologic or cell-based approaches, with continuous-delivery systems reviewed separately. **Results:** Intranasal rescue therapies, such as apomorphine, provide rapid improvement during OFF episodes (periods when the effects of PD medications fade and symptoms reappear), but tolerability issues and nasal irritation limit usage. Early-phase studies suggest intranasal delivery enables quick symptom relief and favorable pharmacokinetics, though most trials are small and focus on feasibility. New approaches include antioxidants, neurotrophic factors, gene therapy, and cell-based methods, with advanced formulations enhancing nasal retention and brain uptake. However, more translational evidence and long-term safety data are needed. **Conclusions:** Intranasal therapies for PD offer rapid rescue and expand options beyond standard drugs. Large, well-designed trials are needed to confirm efficacy, long-term safety, and optimal formulations. Intranasal delivery remains an emerging but potentially transformative strategy requiring further clinical research.

## 1. Introduction

Parkinson’s disease is a progressive neurodegenerative disorder that affects movement and many other parts of daily functioning. It is commonly associated with motor symptoms such as bradykinesia, rigidity, tremor, and postural instability. However, many patients also experience non-motor symptoms that affect cognition, mood, and sleep, thereby reducing overall quality of life [1,2]. Oral therapy involving levodopa-based medication is one of the main treatments used for managing Parkinson’s symptoms, but as the disease progresses, many patients develop motor fluctuations, OFF periods, and less predictable medication responses [1,3]. Oral treatment can also be challenging due to swallowing difficulties, delayed gastric emptying, and unpredictable absorption, which can make the timing and magnitude of medication effects less consistent [3,4].

These limitations have led to interest in alternative ways of delivering treatment that may make the effects of medication faster or more consistent. Intranasal delivery is one approach being studied because it can avoid first-pass metabolism and gastrointestinal absorption variability while offering a noninvasive route that may allow faster access to the central nervous system [5]. In Parkinson’s disease, this route has been explored for different purposes, including rapid rescue during OFF periods, antioxidant therapy, metabolic or hormonal treatment, and newer biologic or cell-based approaches. The objective of this review is to analyze clinical trials of intranasal therapies for Parkinson’s disease and organize them by treatment type and therapeutic intent.

This review evaluates clinical evidence on intranasal drug delivery for Parkinson’s disease, focusing on therapeutic purpose, pharmacokinetics, and clinical outcomes. The literature search was conducted from January 6 through July 2026 using PubMed and ClinicalTrials.gov, with no restriction on publication year. Search terms included combinations of “Parkinson’s disease,” “intranasal,” “nose-to-brain delivery,” “apomorphine,” “glutathione,” “insulin,” “NeuroEPO,” “levodopa-carbidopa,” “stem cells,” “stromal cells,” and “foslevodopa/foscarbidopa.” The review includes human clinical trials, pilot studies, pharmacokinetic investigations, and registered trials involving intranasal therapies in Parkinson’s disease. Preclinical studies were evaluated separately, while non-Parkinson’s disease studies were excluded from the main clinical analysis. For translational context, continuous delivery approaches, such as levodopa–carbidopa intestinal gel and continuous subcutaneous foslevodopa/foscarbidopa, are also discussed as comparators because they share the broader goal of reducing motor fluctuations and improving treatment consistency. Studies are organized by treatment class, route of administration, and clinical objective. Intranasal strategies are grouped into rescue, antioxidant, metabolic or hormonal, and biologic or cell-based therapies, while continuous delivery systems are addressed separately to compare their role with intranasal approaches.

## 2. Traditional Treatment Approaches and Rationale for Intranasal Delivery

Traditional Parkinson’s disease treatments primarily focus on improving symptoms by enhancing dopamine signaling in the brain. Oral dopaminergic therapy—most notably levodopa combined with carbidopa—remains the gold standard for controlling motor symptoms, as levodopa is converted to dopamine [1,3]. Additional medications, including dopamine agonists and drugs that extend dopaminergic activity, may also be considered based on a patient’s symptoms and disease stage [1]. While these therapies are often effective, they do not halt disease progression, and their benefits may become less reliable as Parkinson’s advances.

**Figure 1:**
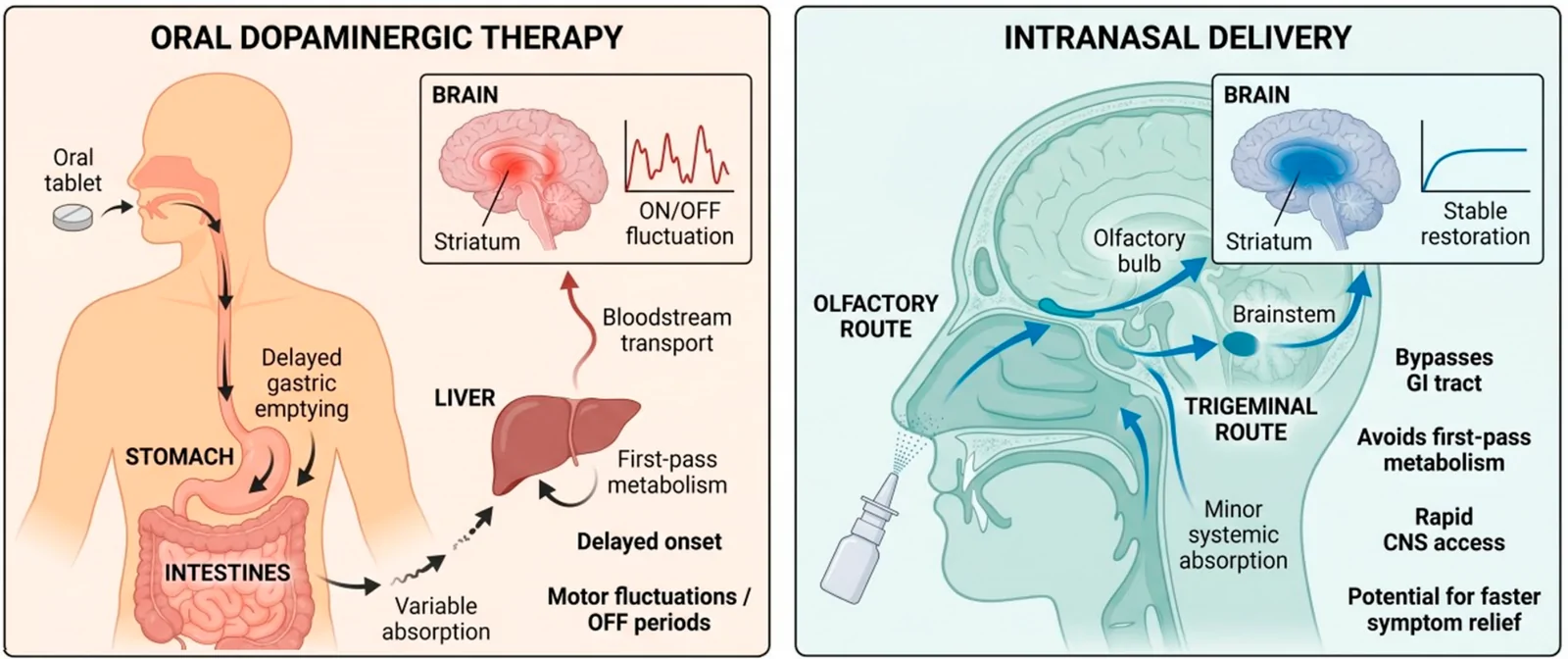
Schematic comparison of oral dopaminergic therapy and intranasal treatment strategies in Parkinson’s disease.

One major drawback of oral therapy is that its effectiveness can be compromised by swallowing difficulties, delayed gastric emptying, first-pass metabolism, and inconsistent gastrointestinal absorption [3,4]. These issues often lead to delayed or unpredictable symptom control, particularly in advanced Parkinson’s disease (Figure 1). As a result, intranasal delivery is being explored as a noninvasive alternative, offering the potential to bypass gastrointestinal absorption and first-pass metabolism. This route may also enable faster access to the central nervous system via olfactory and trigeminal pathways [5]. In Parkinson’s disease, intranasal administration has been investigated for multiple therapeutic purposes, including rapid symptomatic rescue, antioxidant delivery, metabolic or hormonal interventions, and even biologic or cell-based therapies.

## 3. Intranasal Rescue Therapy

**Figure 2:**
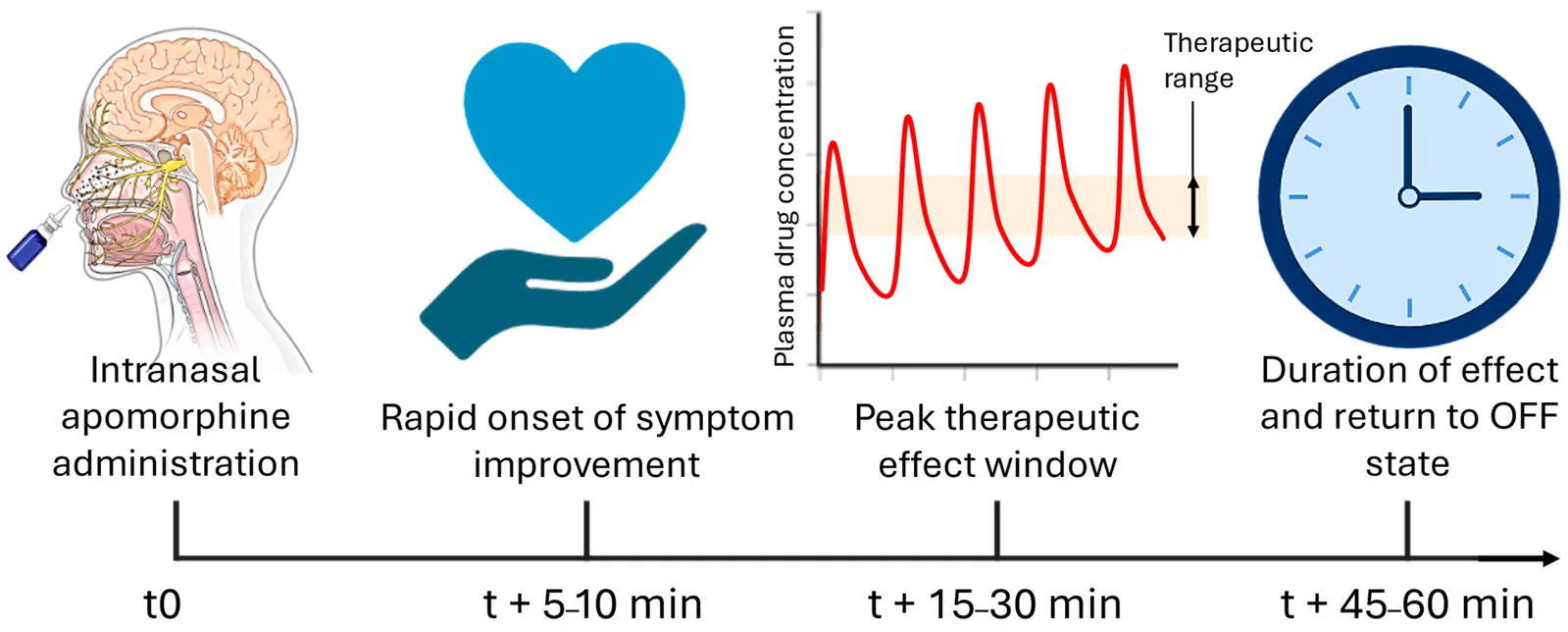
Timeline of OFF-to-ON rescue with intranasal apomorphine in advanced Parkinson’s disease. When administered during an OFF episode (t0), intranasal apomorphine produces rapid dopaminergic motor improvement, with ON typically achieved within 5–10 min. Peak effects occur between 15–30 min, and benefits may last up to 45–60 min before symptoms revert toward OFF. Data points summarize results from open-label and controlled studies [6,7,8].

Rescue therapy in Parkinson’s disease refers to a treatment taken as needed to rapidly relieve symptoms during an OFF episode, rather than a scheduled medication for ongoing symptom control (Figure 2). The primary goal of rescue therapy is to quickly transition the patient from an OFF state back to an ON state. Intranasal apomorphine serves this role effectively, as it is a dopamine agonist that directly stimulates dopamine receptors, thereby offering rapid symptom improvement [6,7]. The intranasal route is particularly valuable for rescue therapy because it can deliver medication more quickly and conveniently during sudden OFF episodes, when oral medications may take too long to work [3,5]. Therefore, studies of intranasal apomorphine focus on the speed of symptom relief, the duration of response, and the tolerability of side effects such as nausea, orthostatic hypotension, or nasal irritation [6,7,8].

Early clinical studies evaluated whether intranasal apomorphine could effectively reverse OFF episodes in patients with levodopa-induced motor fluctuations (Table 1). In an open-label trial involving 11 patients, intranasal apomorphine produced a rapid OFF-to-ON transition, comparable to each patient’s standard levodopa/carbidopa response among study completers. The key benefit was its fast onset, typically within 10 min. However, tolerability was a significant limitation, with side effects such as nausea, vomiting, and one instance of orthostatic hypotension, with one patient withdrawing due to side effects despite anti-nausea medication [6].

Further evidence of the rapid nasal route comes from a small pharmacokinetic study comparing intranasal and subcutaneous apomorphine in seven patients with ON–OFF symptoms. The study found that intranasal administration resulted in rapid absorption, with timing comparable to the injection route, supporting its potential as a fast rescue option. However, because the study focused on blood levels rather than clinical outcomes, it primarily demonstrates the practicality and speed of the nasal route, without confirming clinical benefit [8].

Stronger evidence comes from a small double-blind, placebo-controlled crossover study involving nine patients with advanced, levodopa-responsive Parkinson’s disease. In this study, intranasal apomorphine—but not placebo—improved motor performance during in-person clinical assessments, with results indicating a rapid onset and short duration, consistent with rescue therapy. However, tolerability remained a significant limitation, particularly due to frequent, sometimes severe, nasal irritation. These side effects posed practical challenges despite the medication’s apparent effectiveness. Overall, intranasal apomorphine currently offers the strongest symptomatic rescue rationale among intranasal therapies studied for Parkinson’s disease, but side effects and nasal tolerability remain key barriers to broader use [7].

## 4. Intranasal Antioxidant Therapy

**Figure 3:**
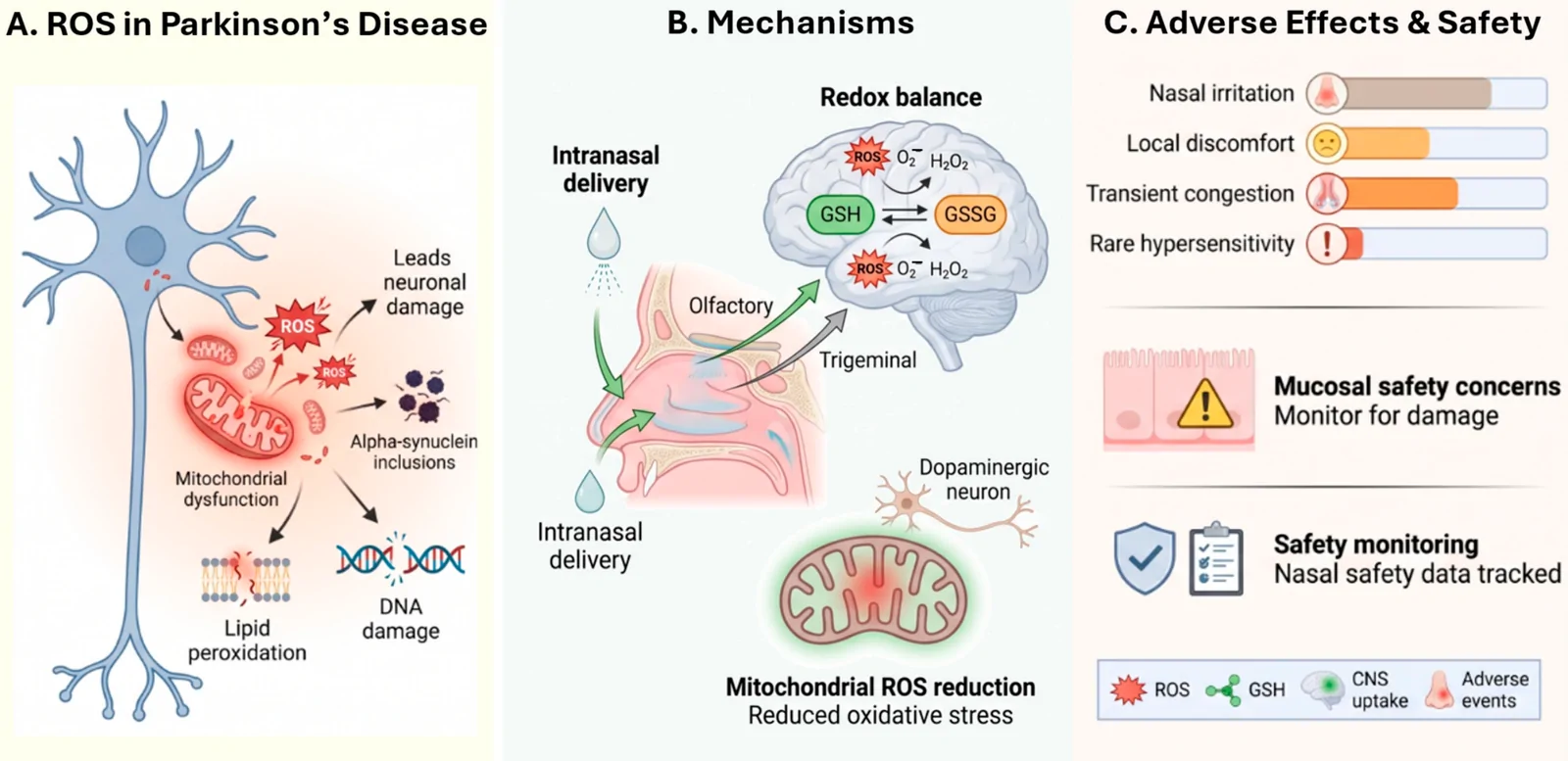
Mechanistic rationale, delivery pathway, and safety profile of intranasal antioxidant therapy in Parkinson’s disease. Panel (**A**) highlights ROS-driven neuronal damage from mitochondrial dysfunction and lipid peroxidation. Panel (**B**) outlines the intranasal delivery mechanism of reduced glutathione (GSH) with CNS uptake, redox balance (GSH/GSSG), and downstream mitochondrial ROS reduction. Panel (**C**) summarizes safety and tolerability issues, including nasal irritation, local discomfort, transient congestion, and safety monitoring considerations, with icons for mucosal safety and adverse event tracking. Together, the panels illustrate the initial studies on GSH primarily evaluated its safety and tolerability.

Intranasal antioxidant therapy is based on the hypothesis that oxidative stress contributes to neuronal injury in Parkinson’s disease (Figure 3). As one of the body’s primary antioxidants, reduced glutathione (GSH) has been investigated as a potential means of maintaining redox balance and mitigating oxidative damage [9,10]. The intranasal route is noteworthy because it offers a noninvasive approach to determine if GSH can access or influence the central nervous system—an essential factor in evaluating antioxidant therapies for Parkinson’s disease [5]. These studies are significant not only for assessing whether GSH can improve symptoms, but also for determining if intranasal delivery is safe, well-tolerated, and capable of producing measurable biological effects in the brain.

In a randomized, double-blind Phase I/IIa trial, Mischley et al. administered intranasal reduced GSH to 30 patients with Parkinson’s disease, assigning them to placebo, 300 mg/day, or 600 mg/day groups for three months. Results indicated that intranasal GSH was generally safe and well-tolerated, with no significant safety concerns observed during treatment. While the study’s small size and design precluded definitive conclusions about clinical efficacy, its main value was in demonstrating that repeated intranasal dosing is feasible and safe for further investigation in larger trials [9].

The subsequent randomized, double-blind, placebo-controlled Phase IIb trial investigated whether intranasal GSH resulted in greater improvements in Parkinson’s symptoms than placebo. In this study, 45 participants with Hoehn and Yahr stage 1–3 Parkinson’s disease were assigned to receive placebo, 100 mg, or 200 mg of GSH three times daily for 12 weeks, followed by a 4-week washout period. Although the higher-dose group showed improvement from baseline on the Unified Parkinson’s Disease Rating Scale (UPDRS), neither GSH dose was clearly superior to placebo in the primary outcome. All groups, including the placebo group, improved during treatment, complicating efforts to distinguish genuine treatment effects from placebo response or natural symptom fluctuations. While this Phase IIb trial supports continued research into intranasal GSH, it did not provide clear evidence of symptomatic benefit over placebo [10].

A related biomarker study assessed whether intranasal GSH could result in measurable uptake in the central nervous system. Mischley et al. utilized magnetic resonance spectroscopy to monitor brain GSH levels after intranasal administration in patients with Parkinson’s disease. This approach advanced research beyond symptom assessment by directly evaluating the potential of intranasal GSH to influence a biochemical marker in the brain. While this biomarker study does not establish clinical efficacy, it is valuable in determining whether intranasal GSH affects the central nervous system. Collectively, the available studies support ongoing exploration of intranasal antioxidant therapy, though conclusive symptomatic benefits in Parkinson’s disease have not yet been demonstrated [11].

## 5. Intranasal Metabolic and Hormonal Therapy

**Figure 4:**
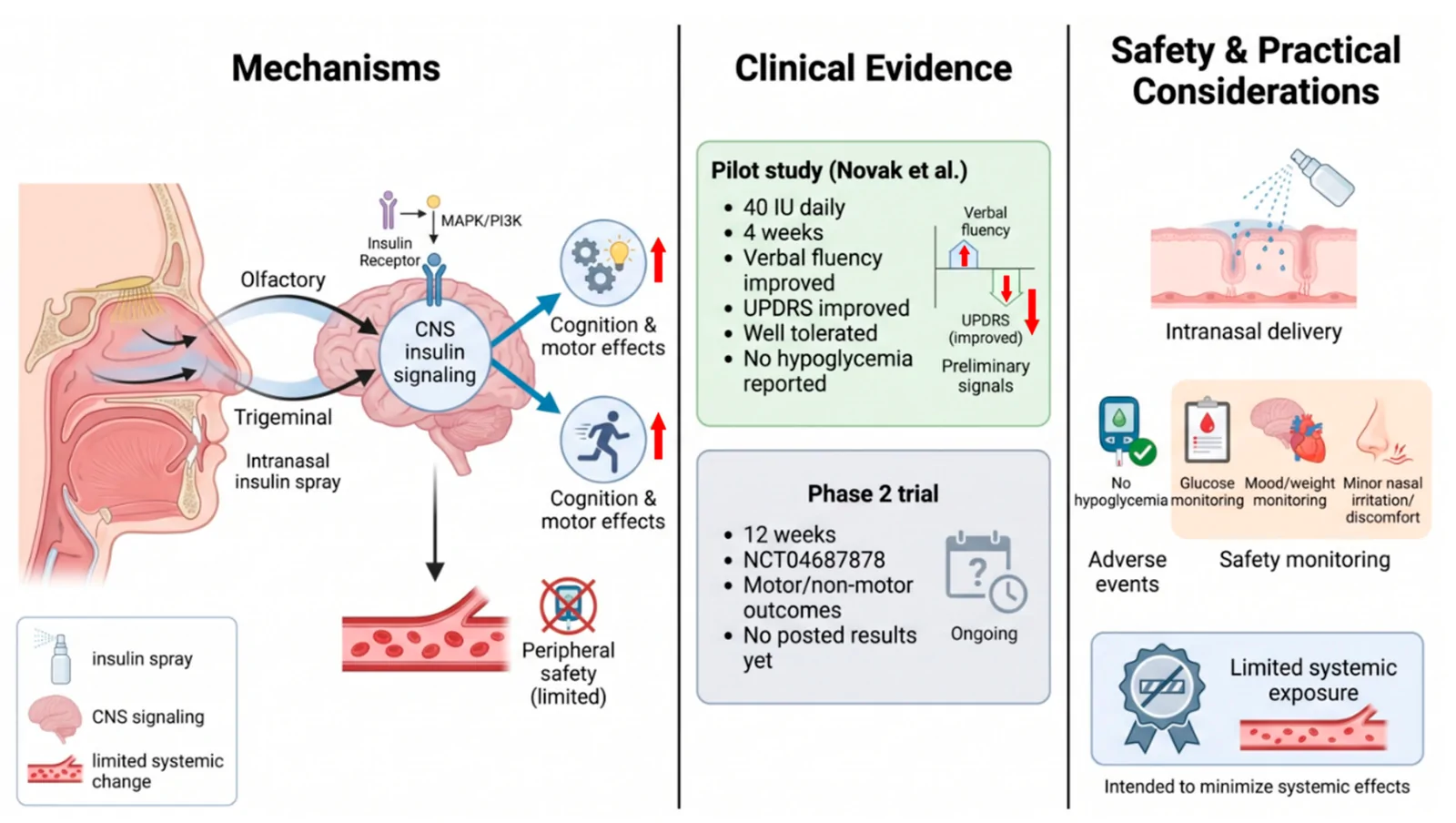
Intranasal insulin delivery and central insulin signaling in Parkinson’s disease. Panel (**left**) illustrates intranasal delivery to the brain via olfactory/trigeminal pathways and the hypothesized central actions of insulin on CNS signaling, cognition, and motor networks, while highlighting the goal of limiting peripheral glucose effects. Panel (**middle**) summarizes early clinical evidence from Novak et al. (pilot study [12]) and a larger Phase 2 trial [13]. Panel (**right**) depicts safety and monitoring considerations, including the lack of reported hypoglycemia in available studies, potential nasal tolerability issues, and the need for ongoing safety surveillance and further exploration in larger, well-designed trials. Red arrows indicate increase or decrease in parameters.

Intranasal metabolic and hormonal therapy differs from antioxidant therapy in that it does not primarily aim to reduce oxidative stress. This section highlights insulin, a hormone commonly associated with blood sugar regulation, but which also exerts significant effects in the brain. In Parkinson’s disease, intranasal insulin has been investigated due to its potential roles in cognition, neuronal signaling, and neuroprotection (Figure 4). Administering insulin intranasally enables more direct delivery to the central nervous system while minimizing systemic side effects, such as alterations in blood glucose levels.

The primary published clinical study on intranasal insulin for Parkinson’s disease was a small, randomized, double-blind, placebo-controlled pilot trial. Novak et al. administered 40 IU of intranasal insulin once daily for four weeks to patients with Parkinson’s disease and one patient with multiple system atrophy [12]. They reported early signs of benefits, including improved verbal fluency compared to baseline and reductions in Hoehn and Yahr severity and UPDRS motor scores within the insulin group. Intranasal insulin was well tolerated, with no cases of hypoglycemia or serious adverse events attributed to the treatment. However, given the study’s limited size and short duration, these results are preliminary and require confirmation in larger, longer-term trials to determine the true efficacy of intranasal insulin in Parkinson’s disease [12].

A larger Phase 2 trial (NCT04687878) also evaluated intranasal insulin in patients with Parkinson’s disease. This randomized, double-blind study compared intranasal insulin to normal saline over 12 weeks, assessing both motor and non-motor outcomes, including MDS-UPDRS scores, cognition, mood, fatigue, and fall risk. Although the trial is marked as completed, the results have not yet been published, so its effectiveness cannot be determined at this time. Current evidence suggests that intranasal insulin remains an early-stage, investigational treatment. While preliminary findings are promising, there is still insufficient data to conclude whether it offers a meaningful benefit for Parkinson’s symptoms [13].

## 6. Intranasal Biologics and Cell-Based Therapy

**Figure 5:**
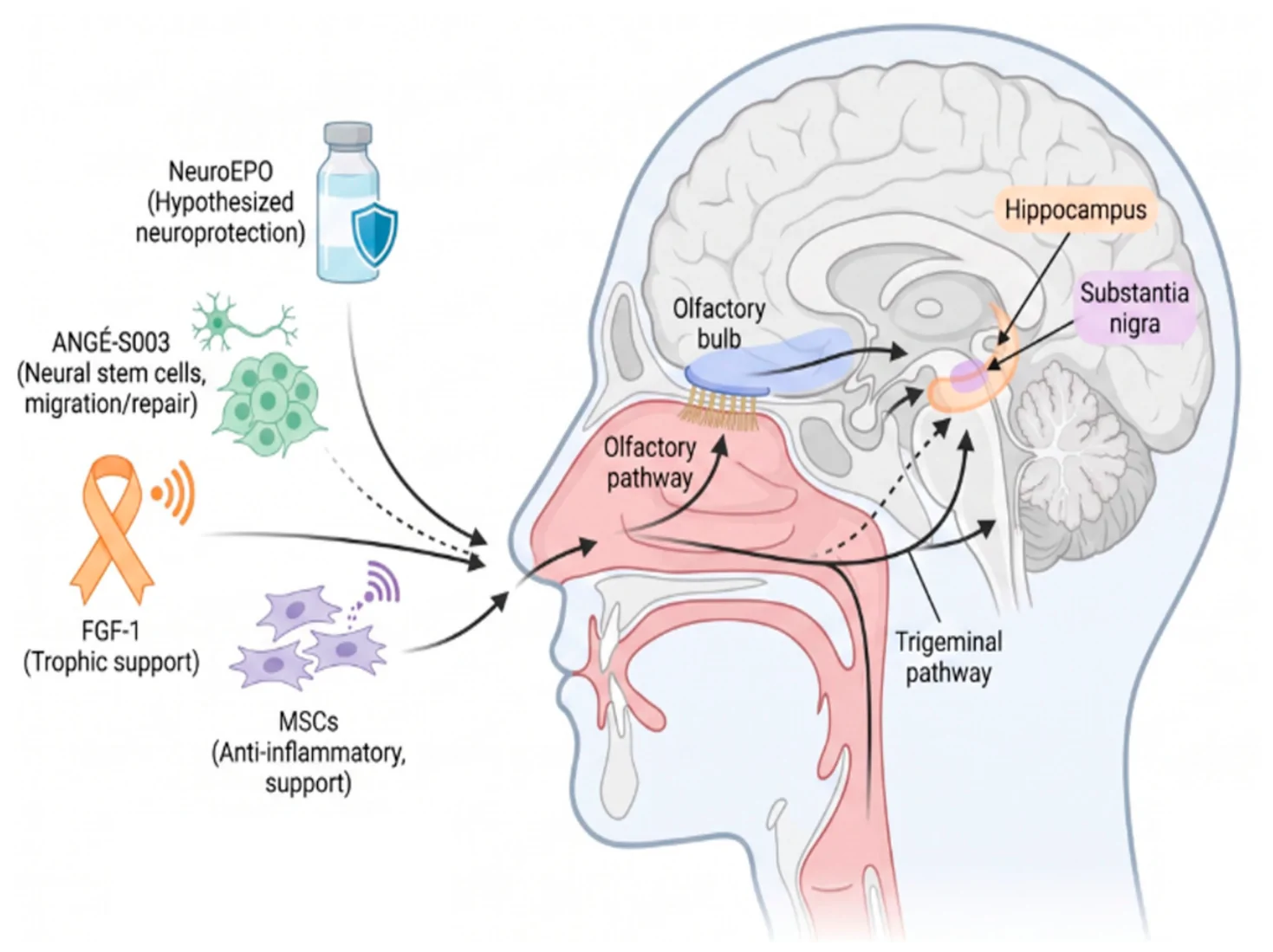
Intranasal delivery pathways for biologic and cell-based therapies in Parkinson’s disease, illustrating NeuroEPO, ANGÉ-S003 neural stem cells, intranasal FGF-1, and stromal/mesenchymal cells. Pathways from the nasal cavity to the brain via olfactory and trigeminal routes, with target regions and potential mechanisms (neuroprotection, anti-inflammatory effects, repair). Emphasis on early-phase safety, dose escalation, and MRI safety monitoring.

Intranasal biologics and cell-based therapies represent a new frontier in Parkinson’s disease treatment. While previous approaches in this review focus on rapid symptom relief, antioxidant support, or metabolic and hormonal modulation, biologic and cell-based strategies are being investigated for their potential to protect neurons, reduce inflammation, and support neural repair or regeneration (Figure 5). Because this field is still in its early stages, the available evidence consists mainly of safety data and preliminary clinical findings, rather than definitive proof of efficacy.

One example of an intranasal biologic therapy is NeuroEPO, a nasal erythropoietin-based formulation with potential neuroprotective properties. In a study by García-Llano et al., the short-term tolerability of intranasal NeuroEPO was assessed in patients with Parkinson’s disease. The treatment was generally well tolerated, with adverse events reported as mild and transient. However, as the study primarily evaluated short-term safety, it does not establish whether NeuroEPO improves symptoms or alters disease progression [14].

The most advanced intranasal cell-based trial to date is the Phase 1 study of ANGE-S003 human neural stem cells. This 12-month, single-center, open-label, dose-escalation trial involved 18 patients with advanced Parkinson’s disease, each receiving four intranasal transplantations of human neural stem cells at escalating doses. The study’s significance lies in its comprehensive safety assessment, including extended MRI monitoring for abnormal growth or mass formation. No serious adverse events related to ANGE-S003 occurred, and MRI scans revealed no evidence of mass formation. Although some clinical improvement was noted, the small, open-label, non-placebo-controlled design means these results remain preliminary [15].

Several other biologic and cell-based approaches remain in early development. For example, a registered Phase 1 trial (NCT05493462) is investigating intranasal human fibroblast growth factor-1 (FGF-1) in Parkinson’s disease [16]. Additional studies of stromal and mesenchymal stem cells (NCT07232147, NCT06858254) further illustrate the exploration of intranasal delivery for regenerative purposes in Parkinson’s disease [17,18]. As these studies are in preliminary stages or lack published efficacy results, they are best viewed as emerging research directions rather than established treatments.

## 7. Clinical Implications and Current Limitations of Intranasal Therapies

**Figure 6:**
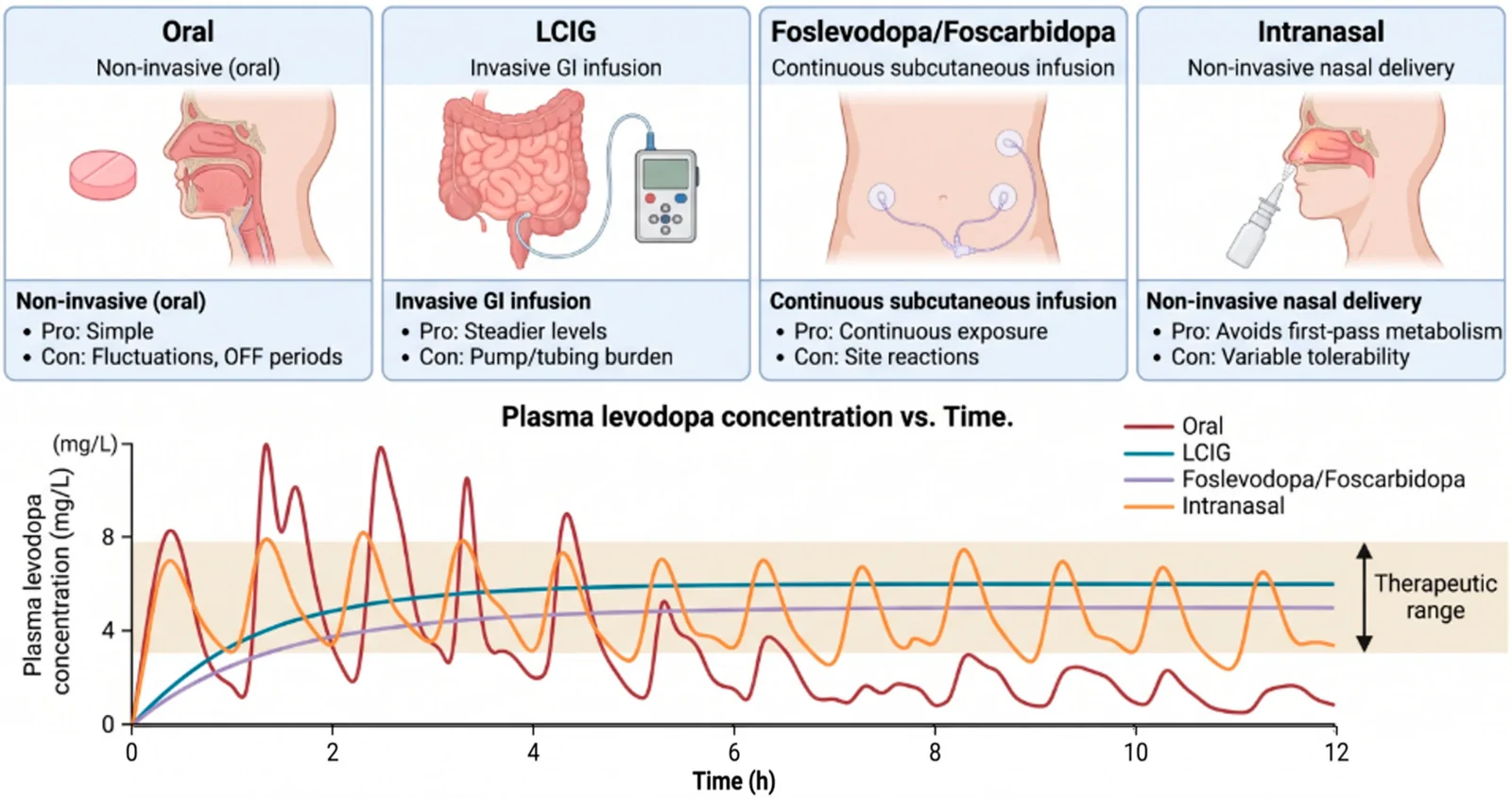
Schematic comparison of drug delivery methods in Parkinson’s disease. Panels show oral tablets, levodopa–carbidopa intestinal gel (LCIG), continuous subcutaneous foslevodopa/foscarbidopa, and intranasal delivery. Each panel displays a typical administration site, invasiveness, and a representative plasma levodopa profile (pulsatile vs. steady). Pros and cons highlight variability, device burden, and practical considerations influencing real-world use. The concentration–time curves shown are hypothetical examples, intended to illustrate typical pharmacokinetic behavior, and do not reflect actual clinical measurements or data from any specific study.

A central challenge in Parkinson’s disease treatment is achieving more consistent medication effects as disease progression leads to increased symptom variability (Figure 6). Oral levodopa remains a cornerstone therapy, but its effectiveness often diminishes over time due to fluctuating responses, OFF periods, and motor complications [3]. While levodopa–carbidopa intestinal gel (LCIG) and continuous subcutaneous foslevodopa/foscarbidopa are not administered intranasally, they are relevant examples because they aim to deliver medication more continuously, thereby minimizing fluctuations and improving symptom control.

LCIG delivers levodopa–carbidopa via continuous intestinal infusion, resulting in more stable levodopa plasma levels than oral tablets while maintaining similar overall drug exposure (Table 2). This steadier delivery helps mitigate motor fluctuations in advanced Parkinson’s disease [19,20]. Similarly, continuous subcutaneous foslevodopa/foscarbidopa provides a 24 h infusion through the skin, targeting the same issue of fluctuating responses. Phase 3 studies have demonstrated that foslevodopa/foscarbidopa increases ON time without troublesome dyskinesia and reduces OFF time relative to oral therapy [21]. Patient feedback indicates that the pump system is generally manageable and can improve consistency of symptom control, though device burden—such as pump size, tubing, and infusion-site complications—remains a significant limitation [22]. These cases underscore the importance of evaluating new delivery systems not only for therapeutic efficacy but also for tolerability, ease of use, and patient burden.

Against this backdrop, intranasal therapies offer several potential advantages: they are noninvasive, bypass gastrointestinal absorption variability and first-pass metabolism, and may provide more rapid or direct delivery to the brain [5]. Among intranasal options, the most robust evidence exists for intranasal apomorphine as a rapid-acting rescue treatment for OFF periods. Clinical studies have shown rapid symptom improvement, but practical use has been limited by side effects such as nausea, orthostatic hypotension, and nasal irritation [6,7,8]. Other intranasal agents, such as glutathione and insulin, have shown encouraging preliminary results, but larger trials have yet to confirm consistent clinical benefits. Biologic and cell-based strategies remain in the early stages of investigation and cannot yet be considered established therapies [14,15,16,17,18].

Overall, current evidence suggests that intranasal delivery holds promise but remains in the early stages of clinical development. No intranasal therapy has yet been proven to alter the underlying course of Parkinson’s disease. Most non-rescue strategies are still supported primarily by early-phase safety, tolerability, biomarkers, or small clinical studies. Common limitations include small sample sizes, brief follow-up periods, a lack of robust placebo-controlled data, and variable symptom assessment. Future research should prioritize larger, randomized controlled trials with longer durations, standardized outcome measures, and validated biomarkers to demonstrate target engagement and clinical efficacy. It is also essential to report on device usability, nasal tolerability, patient comfort, and real-world feasibility, as these factors will be crucial for successful clinical adoption.

## 8. Preclinical Investigations of Nose-to-Brain Delivery

Nose-to-brain delivery has emerged as a promising preclinical strategy for central nervous system therapeutics, enabling partial circumvention of the blood–brain barrier and first-pass metabolism through direct transport via the olfactory and trigeminal pathways. This route also offers the advantage of potentially reducing systemic exposure [23]. In Parkinson’s disease (PD), nose-to-brain delivery is particularly notable, as conventional oral and injectable treatments frequently result in variable and suboptimal brain exposure. Additionally, many neurotrophic and nucleic acid-based therapies face significant barriers to reaching affected brain regions effectively.

**Figure 7:**
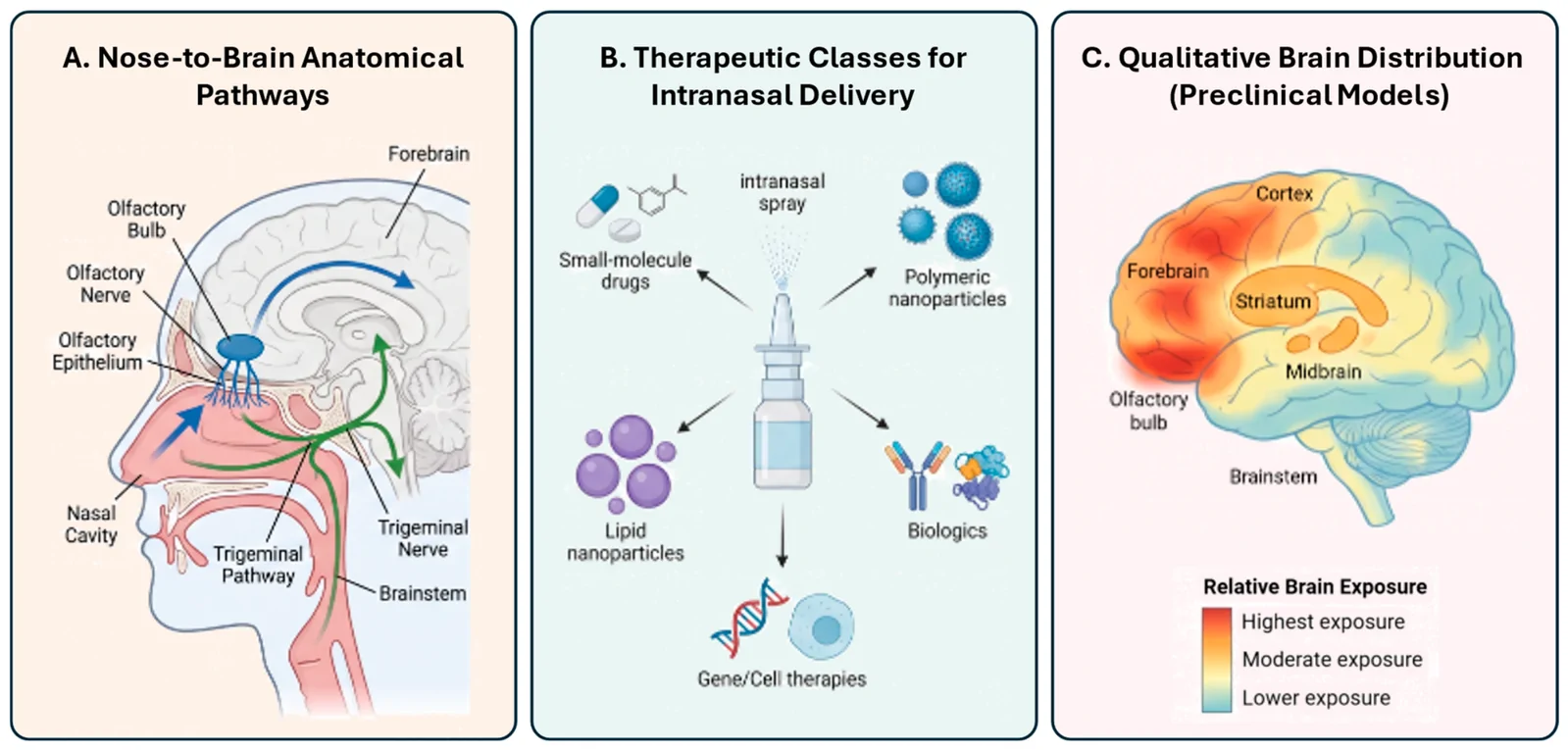
Schematic overview of nose-to-brain delivery routes and brain distribution of representative therapeutic classes for Parkinson’s disease. (**A**) Sagittal view of the nasal cavity and head illustrating the two principal pathways mediating direct nose-to-brain transport: the olfactory route (blue arrows) from the olfactory epithelium via olfactory nerve fibers to the olfactory bulb and forebrain, and the trigeminal route (green arrows) from the nasal epithelium via trigeminal nerve endings to the brainstem and deeper brain regions. (**B**) Representative classes of intranasally delivered agents investigated in preclinical Parkinson’s disease models. (**C**) Qualitative summary of relative brain distribution of intranasally delivered agents in preclinical studies, with color intensity indicating approximate regional exposure: highest in the olfactory bulb and forebrain, moderate in the striatum and midbrain (including substantia nigra), and lower in posterior and deep brainstem regions.

Recent reviews underscore that intranasal delivery in PD models allows for rapid brain access, improved regional targeting, and compatibility with a wide range of therapeutics—including small molecules, proteins, peptides, genes, and cells (Figure 7). Nevertheless, the field remains in the preclinical stage due to persistent challenges such as mucociliary clearance, enzymatic degradation, short nasal residence time, formulation instability, and unresolved questions regarding long-term safety [23,24,25].

Preclinical studies in PD predominantly employ toxin-based rodent models, particularly 6-hydroxydopamine and MPTP systems, to assess whether intranasal formulations enhance brain uptake and improve behavioral or dopaminergic outcomes compared with conventional routes [26,27,28]. Commonly assessed endpoints include striatal dopamine content, preservation of tyrosine hydroxylase, oxidative stress markers, inflammatory responses, and motor outcomes such as rotarod performance, rigidity, akinesia, and gait [29,30,31].

Chitosan- and PLGA-based nanoparticles, as well as mucoadhesive nanoemulsions, rank among the most extensively studied delivery systems. These platforms are designed to prolong nasal residence time, enhance permeation across the nasal epithelium, and protect therapeutic cargo from degradation [32]. For instance, Wen and Ren (2024) demonstrated that intranasally administered selegiline-loaded chitosan nanoparticles improved motor performance and elevated both brain dopamine and neuroprotective markers in PD rat models, highlighting the potential of formulation engineering to enhance the efficacy of established antiparkinsonian drugs [23].

Other preclinical studies have investigated nanoemulsions for drugs such as selegiline, ropinirole, levodopa, ibuprofen, and resveratrol, reporting increased nasal permeability, higher brain concentrations, and improved neurobehavioral or oxidative-stress outcomes compared with suspensions or non-targeted formulations. A common design strategy involves chitosan coating or alternative surface modifications to enhance mucoadhesion and facilitate uptake via the olfactory or trigeminal pathways [33,34].

Solid lipid nanoparticles and nanostructured lipid carriers constitute another key category under investigation. These systems are capable of encapsulating both lipophilic and hydrophilic drugs, offering potential for controlled release and favorable biocompatibility. Recent reviews highlight their promise for delivering agents such as rotigotine, ropinirole, selegiline, rasagiline, various levodopa formulations, and antioxidants. Nonetheless, most studies to date have concentrated on pharmacokinetics and preliminary efficacy, with comprehensive translational validation still lacking [35,36,37].

Hybrid systems that integrate nanocarriers with thermosensitive gels or hydrogels are attracting increasing interest, as they address a major limitation of this route: rapid clearance from the nasal cavity. The recent literature describes examples such as rotigotine polymer-micelle thermosensitive gels, levodopa-loaded chitosan nanoparticles in Pluronic gel, and magnolol nanocrystal hydrogels—all shown to improve nasal retention, brain uptake, or behavioral outcomes in animal models [35].

A key advantage of the nose-to-brain strategy is its applicability beyond conventional dopaminergic drugs. Preclinical PD studies have delivered neurotrophic factors—including brain-derived neurotrophic factor and glial cell line-derived neurotrophic factor—as well as plasmid DNA, antisense oligonucleotides, siRNA nanocarriers, and various stem cell populations, with reported improvements in dopaminergic neuron preservation and motor outcomes in rodents [23,25].

**Figure 8:**
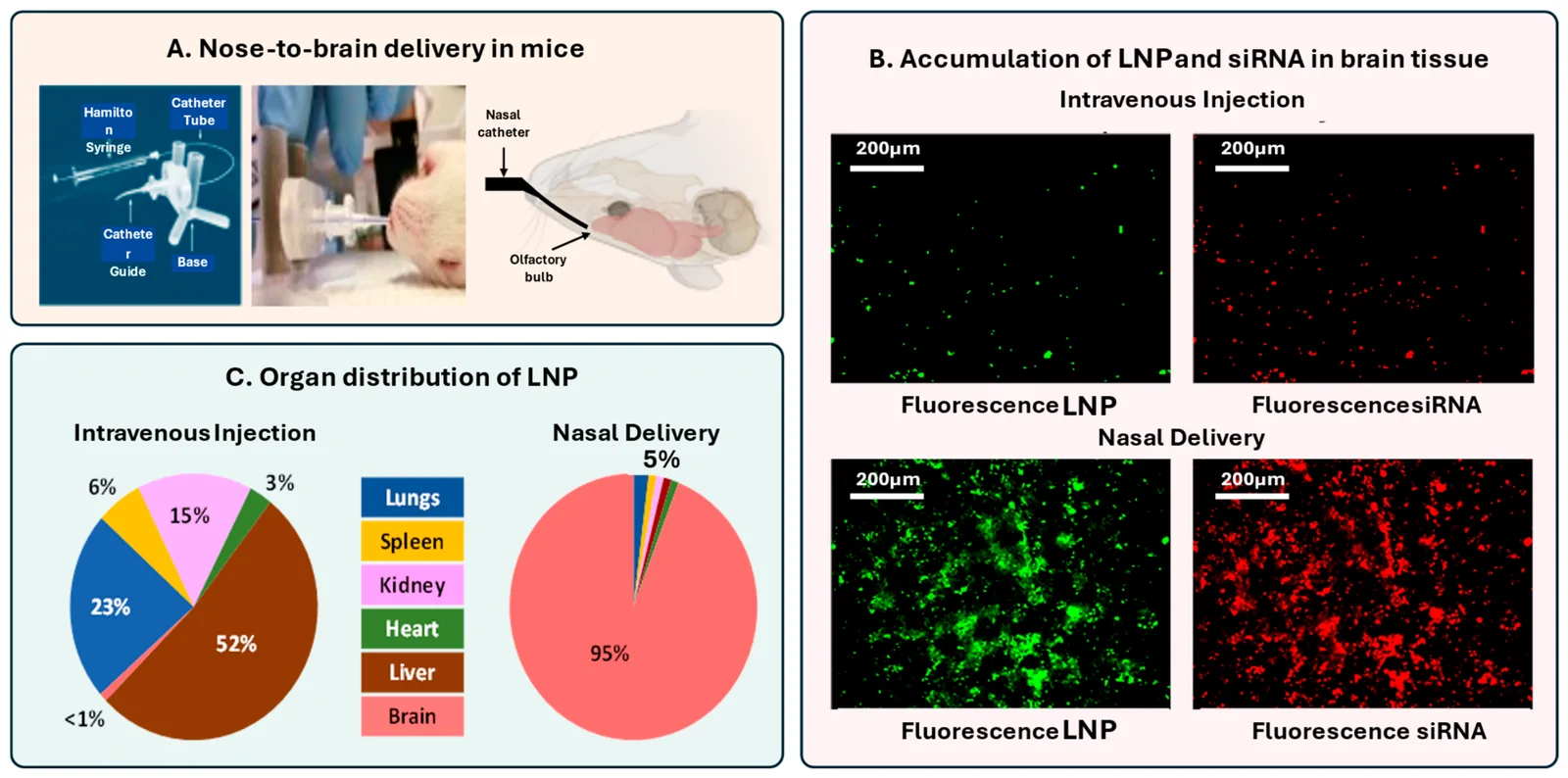
In vivo preclinical evaluation of nose-to-brain delivery of siRNA by lipid nanoparticles (LPN). Redrawn from [37].

Recently, lipid-based nanocarriers have been developed and tested for intranasal siRNA delivery [37,38,39]. These carriers have been shown to efficiently deliver siRNA to brain tissues (Figure 8), with more favorable organ distribution than intravenous administration.

In summary, nose-to-brain delivery in Parkinson’s disease demonstrates significant promise in preclinical studies by bypassing portions of the blood–brain barrier and enabling rapid, widespread brain delivery of diverse therapeutics. However, progress toward clinical translation is hampered by obstacles such as mucociliary clearance, degradation, short nasal residence time, and unresolved safety concerns. In PD models, intranasal administration has improved brain uptake and motor or dopaminergic outcomes, using delivery systems such as chitosan- and PLGA-based nanoparticles, nanoemulsions, lipid nanoparticles, and hybrid gels to enhance cargo retention and protection. The approach leverages olfactory and trigeminal pathways and is being extended beyond dopaminergic drugs to neurotrophic factors, gene therapies, and cell-based strategies, with ongoing efforts focused on translational validation and ensuring long-term safety.

## 9. Future Directions

Future research into intranasal therapies for Parkinson’s disease must advance from small, preliminary studies to larger, rigorously controlled trials. The main goal should move beyond simply demonstrating intranasal delivery, focusing instead on whether these therapies provide consistent and clinically meaningful benefits.

Study designs should be tailored to the specific therapy under investigation. Crossover trials are ideal for rescue therapies, enabling within-patient comparisons during OFF episodes. In contrast, antioxidant, metabolic, biologic, and cell-based therapies benefit from larger, parallel-group trials with longer follow-up periods due to their delayed effects. Primary outcomes should be clearly defined before the study begins to prevent overinterpretation of marginal or uncertain results.

It is essential to include larger and more diverse patient populations. Current research is often limited by small sample sizes, short durations, and homogeneous groups. Conducting multicenter trials allows assessment across different disease stages, symptom profiles, cognitive abilities, and levels of motor fluctuation. Using standardized outcome measures will also improve the comparability of results across studies.

Another key focus should be optimizing dosing and delivery methods. Factors such as formulation, spray volume, device design, dosing schedule, and deposition site within the nasal cavity all affect the efficacy of intranasal therapies. Future studies should directly compare these variables instead of assuming delivery methods are equivalent. This is especially important for insulin, glutathione, and cell-based therapies, where successful outcomes depend on effective delivery to the target area.

Future research should include more robust measures of target engagement. Although clinical scales are valuable, they may not be sufficient on their own. Biomarkers—such as pharmacokinetic profiles, imaging, magnetic resonance spectroscopy, and other objective assessments—can more effectively demonstrate whether therapies reach or affect the central nervous system. These methods are particularly useful when symptom changes are subtle, delayed, or confounded by placebo effects.

Long-term safety monitoring is particularly important for repeated intranasal dosing, growth factors, and cell-based therapies. Trials should monitor for nasal irritation, changes in smell, mucosal injury, immune reactions, and delayed neurological side effects. For neural stem cells, stromal cells, and growth factor approaches, MRI and other structural assessments should be standard components of safety monitoring.

Finally, future research should emphasize patient-centered outcomes. In addition to motor scales such as UPDRS or MDS-UPDRS, studies should assess measures of daily living—such as OFF time, onset of action, cognitive function, fatigue, nasal tolerability, device comfort, and treatment convenience. Consistent reporting of patient adherence is also vital for meaningful comparisons. These factors are vital, since therapy might appear effective in trials but be impractical in real-world use. Comparing intranasal therapies with established standards of care, such as LCIG and continuous subcutaneous infusion of foslevodopa/foscarbidopa, will further clarify their role in the treatment of Parkinson’s disease [19,20,21,22].

In summary, future research should more clearly connect each therapy to its proposed mechanism and clinical use. The ultimate goal should go beyond proving the feasibility of intranasal delivery, aiming instead to establish whether these treatments provide meaningful, reliable, and practical benefits for people with Parkinson’s disease.

## Figures and Tables

**Table 1:** Clinical trials of intranasal apomorphine as rescue therapy for OFF episodes in Parkinson’s disease.

Ref.	Design	Comparator	Main Clinical Findings	Adverse Effects/ Limitations
[6]	Open-label rescue therapy study	Usual levodopa/carbidopa response within the patient	Rapidly relieved OFF states; response similar to usual levodopa/carbidopa	Nausea/vomiting; orthostatic hypotension; 1 dropout; small sample
[8]	Pharmacokinetic study	Subcutaneous apomorphine	Rapid intranasal absorption supported feasibility as a fast rescue route	PK study only; very small sample; limited clinical efficacy data
[7]	Randomized, double-blind, placebo-controlled crossover study	Placebo nasal spray; trimethobenzamide antiemetic also tested	Improved UPDRS motor scores; diary data supported OFF reversal	Nasal irritation; nausea; small sample; tolerability issues

**Table 2:** Comparison of advanced delivery strategies and intranasal therapies in Parkinson’s disease.

Ref	Treatment Approach	Route	Therapeutic Goal	Main Clinical Evidence	Advantages	Limitations/ Patient Experience
[1,3]	Oral levodopa- carbidopa	Oral tablet	Dopamine replacement; Motor symptom control	Established standard symptomatic therapy	Effective for motor symptom control; Widely used	Variable GI absorption; first-pass metabolism; swallowing difficulty; OFF periods; Motor fluctuations in advanced disease
[19,20]	Levodopa–carbidopa intestinal gel (LCIG)	Continuous jejunal infusion	Reduce levodopa plasma variability; Improve motor fluctuations	Steadier levodopa levels than oral tablets; pilot data suggest less OFF time and more ON time without troublesome dyskinesia	More continuous delivery; reduced plasma variability vs. oral dosing	Requires an intestinal tube and a pump; Device burden and tolerability concerns
[21,22]	Continuous subcutaneous foslevodopa/ foscarbidopa	24-h subcutaneous infusion	Continuous levodopa-based delivery; reduce OFF time; improve ON time	Phase 3 trial supports increased ON time and reduced OFF time; patient study reports more consistent control with pump-related challenges	Continuous levodopa-based delivery; avoids intestinal tube placement	Requires infusion device; pump size, tubing, and infusion-site issues may affect use
[6,7,8]	Intranasal apomorphine	Intranasal spray	Rapid rescue therapy for OFF episodes	Open-label, PK, and placebo-controlled studies support rapid OFF-to-ON response and motor improvement	Fast onset; practical rescue option; strongest intranasal clinical evidence to date	Nausea, vomiting, orthostatic hypotension, nasal irritation; small studies
[9,10,11]	Intranasal glutathione	Intranasal reduced glutathione	Antioxidant support; CNS target engagement	Phase I/IIa supports safety; Phase IIb did not show clear superiority over placebo; MRS study evaluated CNS uptake	Supports safety testing and possible CNS target engagement	Clinical benefit inconsistent; not clearly superior to placebo in Phase IIb
[12,13]	Intranasal insulin	Intranasal insulin	Target central insulin signaling; possible cognitive and motor effects	Small pilot suggests early benefit in verbal fluency and some motor/severity measures; Phase 2 completed but results not posted	May affect central insulin signaling while limiting systemic glucose effects	Very small pilot; short duration; Phase 2 efficacy cannot yet be judged
[14,15,16,17,18]	Intranasal biologics and cell-based therapies	Intranasal biologics, growth factors, neural/stromal stem cells	Possible neuroprotective, anti-inflammatory, and regenerative effects	NeuroEPO tolerance study; ANGE-S003 Phase 1 neural stem cell study; FGF-1 and stromal/MSC trials registered	Expands intranasal delivery into biologic and regenerative applications	Mostly early safety and feasibility data; efficacy not established

CNS = central nervous system; FGF-1 = fibroblast growth factor-1; GI = gastrointestinal; LCIG = levodopa–carbidopa intestinal gel; MRS = magnetic resonance spectroscopy; MSC = mesenchymal stem cell; OFF = medication “off” state; ON = medication “on” state; PK = pharmacokinetics.

## Data Availability

Data sharing is not applicable to this article, as no new datasets were generated or analyzed in the current study. This review is based exclusively on previously published literature, which is cited in the reference list.

## Abbreviations

The following abbreviations are used in this manuscript:

CNS	Central nervous system
FGF-1	Fibroblast growth factor-1
GI	Gastrointestinal
GSH	Reduced glutathione
GSSG	Oxidized glutathione
LCIG	Levodopa–carbidopa intestinal gel
MDS-UPDRS	Movement disorder society-unified Parkinson’s disease rating scale
MRS	Magnetic resonance spectroscopy
MSC	Mesenchymal stem cell
OFF	Medication “off” state
ON	Medication “on” state
PD	Parkinson’s disease
PK	Pharmacokinetics
PLGA	Poly(lactic-co-glycolic acid)
ROS	Reactive oxygen species
UPDRS	Unified Parkinson’s disease rating scale
